# Visible light activated SnO_2_:Dy thin films for the photocatalytic degradation of methylene blue

**DOI:** 10.1039/d3ra05424a

**Published:** 2023-10-24

**Authors:** M. Mezyen, G. El Fidha, N. Bitri, F. Harrathi, I. Ly, E. Llobet

**Affiliations:** a Université de Tunis El Manar, Ecole Nationale d'Ingénieurs de Tunis, Laboratoire de Photovoltaïque et Matériaux Semi-conducteurs 1002 Tunis Tunisia marwenmezyen@gmail.com; b Ecole Nationale Supérieure d'ingénieurs de Tunis (ENSIT) Avenue Taha Hussein Montfleury Tunis 1008 Tunisia; c Centre de Recherche Paul Pascal (CRPP) – UMR 5031 115 Avenue Albert Schweitzer 33600 Pessac France; d Universitat Rovira i Virgili, MINOS, ETSE Avda. Països Catalans Tarragona 2643007 Spain eduard.llobet@urv.cat

## Abstract

This paper explores the impact of dysprosium (Dy) doping on structural, optical, and photocatalytic properties of tin oxide (SnO_2_) thin films fabricated *via* spray pyrolysis. Dysprosium doping levels ranged from 0 to 7 at%, and films were grown on glass substrates at 350 °C. X-ray diffraction (XRD) analysis revealed an increase in crystallite size with Dy doping, signifying improved crystalline quality. Simultaneously, dislocation density and strain decreased, indicating enhanced film quality. Texture coefficient (*T*_c*hkl*_) results showed a predominant crystal orientation along the (110) plane due to Dy doping. Optical band gap energy (*E*_g_) decreased with Dy doping up to 5%. Urbach energy increased with Dy doping, suggesting atomic structural flaws and defects. Scanning electron microscopy (SEM) analysis revealed the presence of numerous micro-aggregates on the film's surface. Notably, the density of these micro-aggregates increased proportionally with higher Dy doping levels, particularly emphasizing the pronounced effect observed in SnO_2_:Dy 5% thin films. These findings underscore the potential of Dy-doped SnO_2_ thin films for advanced photocatalytic applications, with SnO_2_:Dy 5% exhibiting favorable properties and demonstrating a 90.99% degradation efficiency in three hours under solar irradiation.

## Abstract 2

This paper explores the impact of dysprosium (Dy) doping on structural, optical, and photocatalytic properties of tin oxide (SnO_2_) thin films fabricated *via* spray pyrolysis.

## Graphical abstract

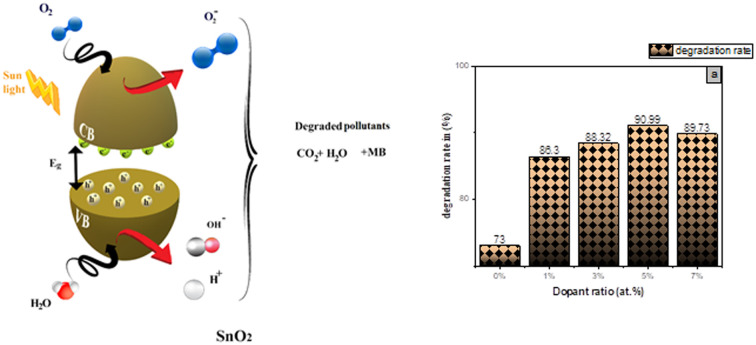

## 1 Introduction

Dyes encompass a category of colored aromatic organic compounds known for their light-absorbing properties, which contribute to their ability to introduce color into the visible spectrum.^1,2^ Various industries, including textiles, food, rubber, printing, cosmetics, medicine, plastics, concrete, and the paper industry, employ a wide range of dyes for various purposes.^3^ It is estimated that there are over 100 000 commercially utilized dyes globally, with an annual production volume ranging from approximately 7 × 10^8^ to 1 × 10^9^ kilograms.^4^ Unfortunately, these industries produce substantial amounts of wastewater that often contains carcinogenic and toxic dyes, thereby contaminating water sources and rendering them unsuitable for human consumption.^5^ Among these sectors, the textile industry stands out as the largest consumer of dyes, with textile dyes being particularly complex compounds featuring diverse structural groups.^10^ Notably, Methylene Blue (MB) is one of the most heavily used substances in the dye industry, finding common application in the coloring of silk, wool, cotton, and paper.^6–8^ MB is classified as an aromatic heterocyclic basic dye.^9^ It is widely recognized as a cationic primary thiazine dye, characterized by its molecular formula C_16_H_18_N_3_ClS and an absorption maximum *λ*_max_ at 663 nm. Remarkably, MB exhibits exceptional water solubility, forming a stable solution with water even at room temperature.^10–13^ Therefore, many efforts have been devoted to remove MB dye from waste water including adsorption,^14–16^ chemical precipitation and some biological means but for their limitations. Photocatalysis, an advanced oxidation process, is highly efficient, cost effective; eco-friendly and stable photocatalysts have found their routes through semiconductors in nano forms. Transparent Conductive Oxides (TCOs) such as TiO_2_, SnO_2_, ZnO, Fe_2_O_3_ and CdO, have been proven to be suitable photocatalysts for the removal of organic water pollutants.^17–19^ Among all these and other photocatalysts, tin oxide (SnO_2_) has gained more attention due to its numerous properties including high charge carrier mobility, a wide band gap, excellent transparency, ease of tunability, as well as remarkable thermal and chemical stability.^20^ Enhancing the photocatalytic performance of SnO_2_ has been a subject of study, with significant improvements achieved through doping with transition metals or their oxides such as Cu,^21^ Ni, Sb,^22^ Co, Fe, and Mg.^23^ This modification has been shown in the literature to influence shifts in optical absorption, elevate surface defect levels, increase the generation of surface oxygen vacancies, and impede charge carrier recombination.^24–27^ Recently, rare earth (RE) metals have been explored as additives due to their interactive electron configuration, excellent catalytic properties and fast oxygen ion mobility, which tend to enhance the speed of degradation by making up for the shortcomings in the structure of the thin films. These interesting materials involve a wide range of elements including Ce,^28^ Y,^29^ Tm,^30^ Tb,^31^ Nd^32^ and Pr.^33^ Numerous studies have investigated the impact of doping with rare earth elements on the structural and photocatalytic properties of thin films. For instance, Loyola *et al.*^34^ explored the synergistic effect of terbium (Tb) doping on SnO_2_ thin films, prepared through nebulized spray pyrolysis and demonstrated an impressive 85% degradation efficiency for 10^−5^ M methylene blue (MB) dye aqueous solution. Similarly, Ayadi *et al.*^35^ examined the influence of cerium (Ce) doping on SnO_2_ thin films, also prepared using spray pyrolysis, and achieved a noteworthy 19.10% degradation of MB dye under UV irradiation. Their results highlighted that the photocatalytic performance was significantly enhanced by the presence of a mixture of tetragonal and cubic phases, particularly with a 2 wt% Ce doping. These two exemplary studies illustrate the profound impact of rare earth element doping on thin films' photocatalytic capabilities and provide valuable insights into the optimization of such materials for efficient dye degradation. Dysprosium (Dy), being among the most abundant rare earth elements, exhibits high surface reactivity, rapidly oxidizing in the presence of air and readily reacting with water to release dihydrogen. Introducing Dy-doping into TCOs has been observed to enhance visible light absorption and suppress the recombination of photo-generated electrons and holes, thus making it a favorable choice for photocatalytic applications.^36^ In this study, Dy-doped SnO_2_ thin films were prepared by means of spray pyrolysis. In contrast to many other film deposition processes, this technique is a fairly straightforward and comparatively inexpensive processing approach (low-cost equipment used). It is an incredibly simple method for creating films of any composition. Spray pyrolysis does not require premium chemicals or substrates, and can be used to deposit thick films, porous films, and to create powder. This adaptable method makes it simple to produce multi-layered films. To the best of our knowledge, spray pyrolysis has never been used to develop Dy-doped SnO_2_ thin films for photocatalysis applications. The main goal of this study was to determine how dysprosium dopant affects the structural, morphological, optical, chemical and electrical properties of SnO_2_ thin films, specifically by studying how the use of rare-earth dysprosium as a dopant helps improve the photocatalytic performance of thin films made of SnO_2_ by focusing on the effects of time, dye concentration, and sample composition on the degradation efficiency of MB.

## 2 Materials and methods

### 2.1 Preparation of the thin films

**Fig. 1:**
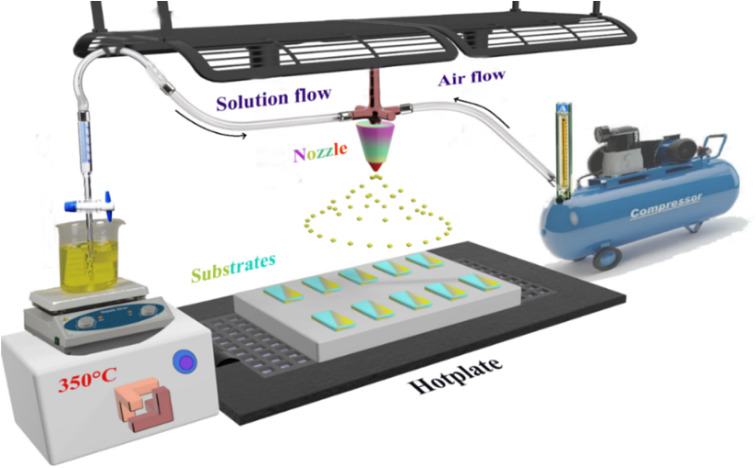
Spray pyrolysis method.

Several separate steps were performed prior to the spraying stage. The glass substrate was treated following a specific cleaning protocol to eliminate impurities using detergent, ionized water, acetone and 1/4 HNO_3_ + 3/4 HCl. The substrate was then dried in an oven at a temperature of 120 °C. The dysprosium (Dy) doped and undoped SnO_2_ thin layers were prepared using a chemical spray pyrolysis technique on a glass substrate heated to a fixed temperature of 350 °C as presented in Fig. 1. Meanwhile, the SnO_2_ solution was prepared by dissolving 0.05 M of tin(ii) chloride SnCl_2_ (99.9%, Sigma-Aldrich) in 40 mL of distilled water. For the doped thin films, the dysprosium DyCl_3_ (DyCl_3_·2H_2_O, Sigma-Aldrich) was mixed with the precursor before being dissolved in concentration ratios of 1, 3, 5 and 7 at%. Since SnO_2_ is insoluble in water, it must undergo continuous magnetic stirring prior to and throughout the entire deposition period with heating to just 30 °C, and with the addition of a few drops of HCl. Tin oxide thin film deposition on a glass substrate is anticipated to occur after the chemical reaction as:

1 SnCl_2_+ H_2_O → SnO +2HCl

2 SnO + 1/2O_2_ → SnO_2_

The solution was sprayed for 10 min at a solution flow of 4 mL min^−1^. All the parameters were controlled, including air compression carrying gas at a fixed flow of 10 mL min^−1^ and substrate temperatures maintained at 350 °C through a thermocouple connected to the hotplate on which the substrates were placed. The space between the nozzle and the substrates was estimated to 20 cm.

All these conditions were designed to promote the formation of the thin layer by maintaining contact between droplets of the solution and the substrates.

### 2.2 Characterization of the thin films

The thin films obtained in the spraying process were then characterized. Their structural properties were determined using X-ray diffraction (XRD) with a Philips X'pert diffractometer with Cu Kα radiation (*λ* = 1.5406 Å) granting a diffraction angle (2*θ*) ranging between 20° and 70°. Optical properties like transmittance and reflectance were obtained using a spectrophotometer (Shimadzu-UV 1800) providing a scan range of between 300 and 1800 nm.

While the measurement of electrical properties such as resistance and resistivity were taken by means of the four-point technique using a Jandel four-point probe head, the factor shape of which is widely known, coupled to an HP 3458A multimeter (Agilent). Field emission scanning electron microscopy was used to examine the surface morphology of the synthesized thin films (FE-SEM JEOL JSM-7800 F).

### 2.3 Photocatalytic tests

The photocatalytic efficiency of pure and Dy-doped (1, 3, 5, and 7% at.) SnO_2_ layers was tested using methylene blue dye (MB). Degradation occurred under solar irradiation (temperature: 35 °C, latitude: 11.24°, northern Tunisia). The photodegradation of MB was conducted using an estimated power level in the range of 180 to 230 W m^−2^. Each of the 5 thin layers were placed in a 40 mL glass container and totally immersed in methylene blue aqueous solution at different concentrations of 2, 3 and 4 mg L^−1^. Next, pure and Dy-doped SnO_2_ layers (1%, 3%, 5%, and 7% at.) were magnetically swirled for 30 minutes, and then left in the dark. This process ensured the balanced adsorption and desorption of the photocatalyst and the dye. Lastly, the samples and solution were exposed to solar irradiation for three hours, with a 3 mL sample taken each hour.

The residual content was determined by a degradation rate *X* defined as follows:^37^3
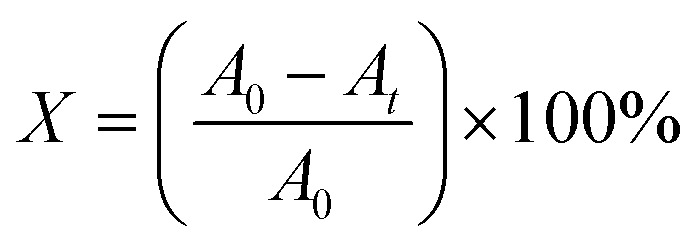
where *A*_0_ is the initial absorbance value and *A*_*t*_ is the Value of the real-time absorbance of measurement at maximum wavelength of the dye.

## 3 Results and discussion

### 3.1 Structural properties: XRD analysis

**Fig. 2:**
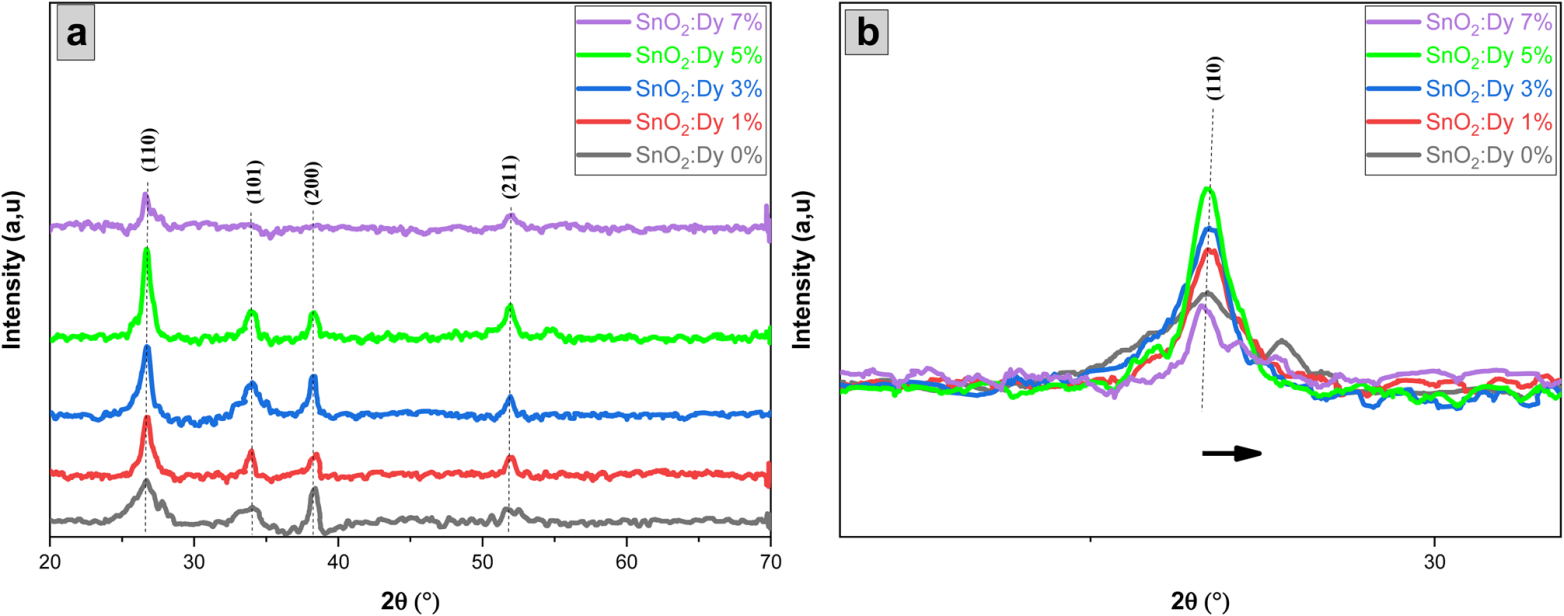
(a) XRD pattern of sprayed undoped and Dy-doped [1, 3, 5 and 7% at.] SnO_2_ thin films, (b) shifting of the main diffraction angle with increasing doping concentrations.

The XRD patterns of undoped and Dy-doped SnO_2_ [1, 3, 5 and 7% at.] thin films are shown in Fig. 2a. All of the XRD patterns are indexed in agreement with standard JCPDS 00-041-1445. The peaks are well identified at the positions 2*θ* = 26.6, 34.4, 38 and 51.5° corresponding to the (*hkl*) planes (110), (101), (200), and (210) respectively, which confirms the tetragonal rutile structure of the SnO_2_ and SnO_2_:Dy thin films with a preferential direction of (110). In Fig. 2b, the 2*θ* angle corresponding to the peak (110) is depicted. A slight shift towards higher angles is noticeable as the dopant concentration increases. This shift can be attributed to the integration of Dy ions into the SnO_2_ crystal structure.^38^ All the peaks detected in the spectra are almost identical, except for thin layers based on SnO_2_:Dy 7%, where the two peaks at positions 2*θ* = (101) and (200) disappear. This may be caused by the excess presence of the Dy^3+^ ion triggering the incipient destruction of the lattice structures of the SnO_2_. According to Table 1, there is not a big difference between the lattice parameters, which increase slightly with increasing the rate of Dy-dopant. It is worth noting that the absence of any additional phases of Dy peaks like Dy_2_O_3_ in any of the patterns indicates the successful incorporation of the dopant particles in the matrix of the SnO_2_.^39^

**Table 1:** Lattice constants, crystallite size (*D*), dislocation density (*δ*), strain (*ε*) and texture coefficient (*T*_c_) of SnO_2_:Dy thin films

Samples	(*hkl*)	2𝜃(°)	*d*_*hkl*_ (Å)	*a* (Å)	*c* (Å)	*D* (nm)	*δ* (10^−3^) (nm^−2^)	*ε* (10^−3^) (rad)	*V* (nm^3^)	*N*	*T*_c_110__
SnO_2_:Dy 0%	(110)	26.69	3.34	4.71	3.17	6.90	21.00	5.47	70.32	0.05	1.07
SnO_2_:Dy 1%	(110)	26.75	3.34	4.73	3.18	12.59	6.31	2.66	71.15	0.09	1.07
SnO_2_:Dy 3%	(110)	26.84	3.33	4.72	3.17	13.00	5.92	2.75	70.62	0.10	1.08
SnO_2_:Dy 5%	(110)	26.64	3.35	4.74	3.19	14.86	4.53	2.44	71.67	0.11	1.19
SnO_2_:Dy 7%	(110)	26.60	3.35	4.75	3.19	7.00	20.00	4.93	71.97	0.05	1.20

The effect of Dy doping is very clear. When the doping rate increase, the intensity of the peaks also increase, with the maximum effect detected in the SnO_2_:Dy 5% layers. In order to better observe the effect of Dy doping on the thin film structures, several structural parameters should be described such as inter-lattice distances *d*_*hkl*_, lattice parameters (*hkl*), crystallite sizes (*D*) as well as other parameters, which are summarized in Table 1.

The crystallite size (*D*) of pure and Dy-doped SnO_2_ thin films are evaluated using the Debye Scherrer equation:^40^4
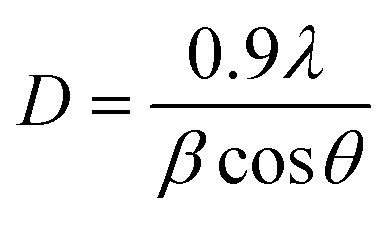
where *λ*, *β* and *θ* are the wavelength of the X-ray diffraction. The full width half maxima (FWHM) of the observed preferential peak are presented in Table 1.

It has been noted that *D* increased with the increase in doping rate up to the highest value of 14.86 nm recorded for the SnO_2_ thin films doped at 5% Dy, suggesting an enhancement in crystalline quality, as shown in Fig. 2.

The dislocation density (*δ*) which provide information about the film's quality and any flaws in the material,^41^ is determined using the eqn (5):^43^5
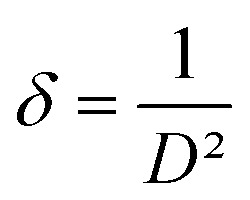


A decrease with the increase of the dopant to reach minimum values of 4.53 × 10^−3^ nm^−2^ for the 5% Dy dopant was observed.^42^

The strain is determined by the eqn (6) below^43^ is tabulated in Table 1:6
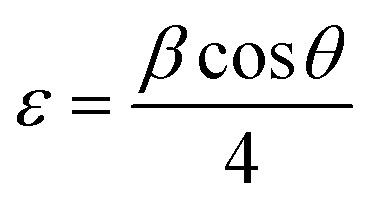


The strain was decreased with the increase of Dy to attend 2.44 × 10^−3^ rad for the 5% thin film, then an increase was subsequently recorded for layers doped to 7%.^44^

The unit cell volume was determined using the eqn (7):

7 *V* = *a*^2^ × *c*

The rise in SnO_2_ crystallite size can be greatly suppressed by doping with Dy, and grain refinement can be accelerated. In addition, the SnO_2_:Dy 5% thin layers yielded the maximum number of unit cells.

The number of unit cells is calculated using the eqn (8):^45^8
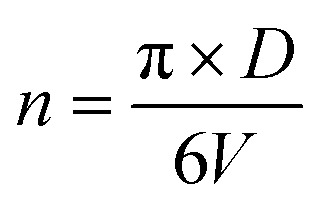


The texture coefficient (*T*_c*hkl*_) which measures the relative degree of preferential orientation of a plane (*hkl*) is determined according to the following formula:^46,47^9
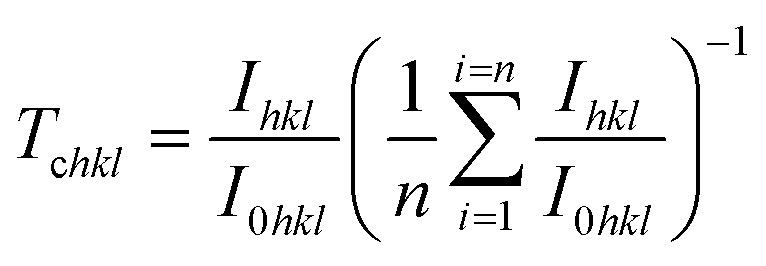
where *n* is the number of diffraction peaks, *I*_0_ is the plane standard intensity (*hkl*) of JCPDS data, *I* is the measured plane intensity (*hkl*) observed in XRD diffractograms.

When *T*_c_ = 1, the crystallites are randomly oriented while the abundance of crystallites in a given plane is indicated by *T*_c_ > 1. If 0 < *T*_c_ < 1, this can denotes the absence of crystallite orientation in this direction.

The texture coefficient for the non-doped and Dy-doped SnO_2_ layers is greater than 1, indicating the predominant tendency of the crystals to be oriented along the (110) plane. The increase in the texture coefficient indicates that the Sn particles have been replaced by the Dy^3+^ ions.^48^

### 3.2 Optical properties

**Fig. 3:**
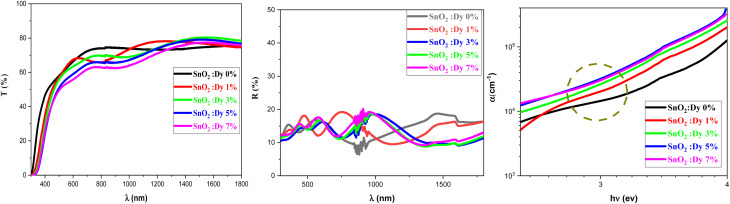
Transmission, reflection and absorption coefficient spectra of SnO_2_:Dy [0, 1, 3, 5 and 7% at.] thin films.

Fig. 3 shows the variation of the transmittance and reflection spectra in the 300–1800 nm wavelength range for the pure and Dy-doped SnO_2_ thin films. As the figure shows, all of the thin films exhibited high transmittance levels, with transmittance reaching up to 80% in the NIR, in good agreement with what could be expected from a (TCO). Transparency decreased slightly as Dy doping increased. The high transparency of these samples would enable their use as an optical window in solar cell applications.

It is important to note the presence of several interference fringes (oscillations with minima and maxima) in the recorded reflection spectra. This is clearly caused by a multiple reflection phenomenon occurring between the free surface and the surface in contact with the substrate. The thickness of the SnO_2_:Dy thin films can be estimated using a common technique, from the locations of the interference reflectance minima and maxima.^49^ The values of the thickness of the SnO_2_:Dy films are illustrated in Table 2, which show that Dy doping increased the thickness of the thin layers. The thickness of thin films made by spray pyrolysis greatly depends on factors such as the concentration of the precursor, the substrate treatment and controlling the spray duration. Doping can generate molecular interactions, which leads to condensed films.

**Table 2:** Thickness, gap energy and Urbach Energy of SnO_2_:Dy thin films

Sample	SnO_2_:Dy 0%	SnO_2_:Dy 1%	SnO_2_:Dy 3%	SnO_2_:Dy 5%	SnO_2_:Dy 7%
Thickness (nm)	239 ± 10	320 ± 10	373 ± 10	380 ± 10	381 ± 10
*E*_g_ (eV)	3.91	3.72	3.63	3.56	3.58
*E*_u_ (meV)	232	458	460	490	526

The reflection rates are between 15 and 20%. In addition, the optical absorption edge of Dy-doped SnO_2_ films is red-shifted compared to pure samples, moving forward to the visible light region. This red shift may be due to the formation of shallow electronic levels within the band gap arising from dopant atoms present in the lattice.^50^

By plotting (*αhν*)^2^ as a function of (*hν*) and based on the Tauc model^51^ indicated in eqn (10), the gap energy was easily estimated the by extrapolating the linear portion of the plots of (*αhν*)^2^*versus hν* to *hν* = 0 and based on the Tauc model interpreted by the following equations:

10 (*αhν*) = *B*(*hν* − *E*_g_)^*n*^

11
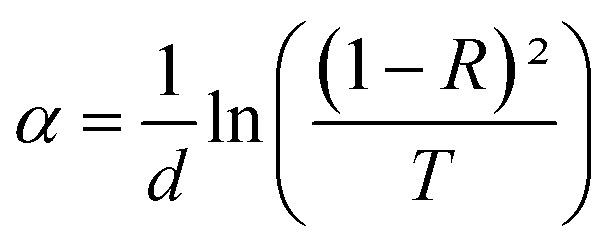
where *ν* is the frequency of the incident photon, *hν* is photon energy, *B* is a constant, *α* is the absorption coefficient and *d* is the thickness of the layers considered.

*E*_g_ is the optical band gap energy presenting the minimum required energy to excite an electron from the valence band to the conduction band and *n* is a fraction whose values depend on the nature of the transition (1/2, 2, 3/2 and 3). In our case, *n* takes 1/2 as a value, which is consistent with direct-allowed transitions.^52,53^

**Fig. 4:**
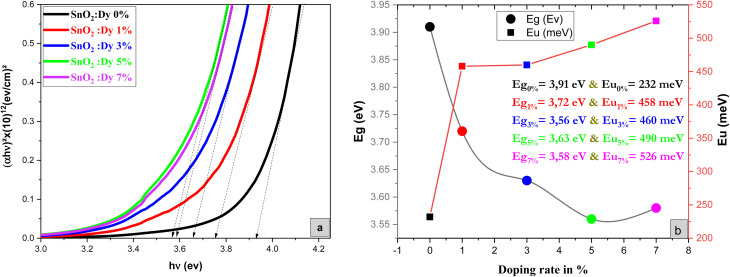
(a) Plot of (*αhν*)^2^*versus* (*hν*) of undoped and Dy-doped SnO_2_ thin films, (b) variation of *E*_g_ and *E*_u_*versus* variation of doping rates of Dy.

As demonstrated in Fig. 4, the presence of a linear portion in the band gap *versus* doping level plot confirms that our material maintains its character as a direct gap semiconductor. The reduction in band gap energy from 3.91 eV to 3.56 eV, observed as the percentage of Dy doping increases up to 5%, can be primarily attributed to the incorporation of Dy ions into the SnO_2_ crystal structure. Dy doping induces lattice distortion and disorder, which, in turn, affects the electronic structure of the material. This distortion results in a reduction of the band gap, as Dy contributes additional energy levels within the band structure. It's important to note that this reduction in the band gap is a characteristic effect of doping and is a well-documented phenomenon in the literature. Beyond the 5% doping level, a slight increase in the band gap is observed. This behavior may be related to the saturation effect of doping, where excessive dopant concentrations can lead to the formation of new phases, lattice defects, or other structural changes, all of which can influence the band gap. These effects are in accordance XRD results.

Meanwhile the increase in the gap in the 7% doped film can be explained by the Burstein-Moss effect, which is an *increase in the absorption edge* generated when the electrons require more energy to go from the valence band to the conduction band when the Fermi level, located within the conduction in the case of *n* type semiconductor, shifts further in the conduction band, resulting in the widening of the band gap of the film.^54^ Palanichamy *et al.* reported similar band gap variations for (Gd^3+^)-doped SnO_2_ thin films prepared with a simplified spray pyrolysis technique using a nebulizer.^55^ We should note that the optical band gap of the film is also known to be effectively changed by defects like impurities, interstitials, oxygen vacancies, and substitutional defects.

This displacement of the energy gap might be in part attributed to the implementation of defects and disorder in the SnO_2_ lattice due to the incorporation of dopants, which has the effect of forming localized states and deep levels in the band gap, which allows the general structure of it to change.^56^ The spectral lines depicted in Fig. 3c show the values of the absorption coefficient of the synthesized thin films as a function of the photon energy. Dysprosium doping caused an increase in optical absorption across all layers, which is consistent with the findings of Gnanasekar *et al.*,^57^ who showed that such doping could give rise to changes in the optical properties of CuO, allowing them to be used for applications that involve radiations and chemical reactions. The values of the absorption coefficients were very high with an average value of 10^5^ cm^−1^ for all thin films, giving our thin films the absorbent character they need for good photocatalytic performance.

In semiconducting materials, structural disorder produces localized states, also known as band tails, around the margins of prohibited bands. The Urbach Energy, also known as the Urbach tail, is the exponentially increasing absorption edge located close to the fundamental band gap.^58^ The Urbach Energy can be used to determine the lattice defect concentration because it characterizes the breadth of the localized states in the principal optical band gap. The following relation is used to assess the Urbach tail:12
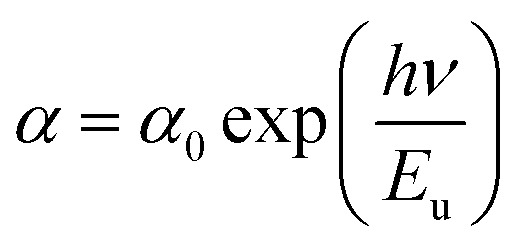
where *E*_u_ stands for the Urbach Energy, *α* is the absorption coefficient, *α*_0_ is a constant and *hν* is the incident photon energy.

Fig. 4b shows that *E*_u_ increased as Dy doping increased, going from 232 to 526 meV, which demonstrates that atomic structural flaws occurred. The increase in the Urbach Energy values with the increase in Dy doping concentration suggests that the band gap structure of the thin films doped with Dy had more defects than before. Actually, the optical data might lead one to believe that Dy atoms were successfully incorporated into the SnO_2_ lattice. The shrinkage of the optical band gap also explains the rise in the Urbach Energy,^59^ as shown in Fig. 4b.

### 3.3 Morphological characterization

**Fig. 5:**
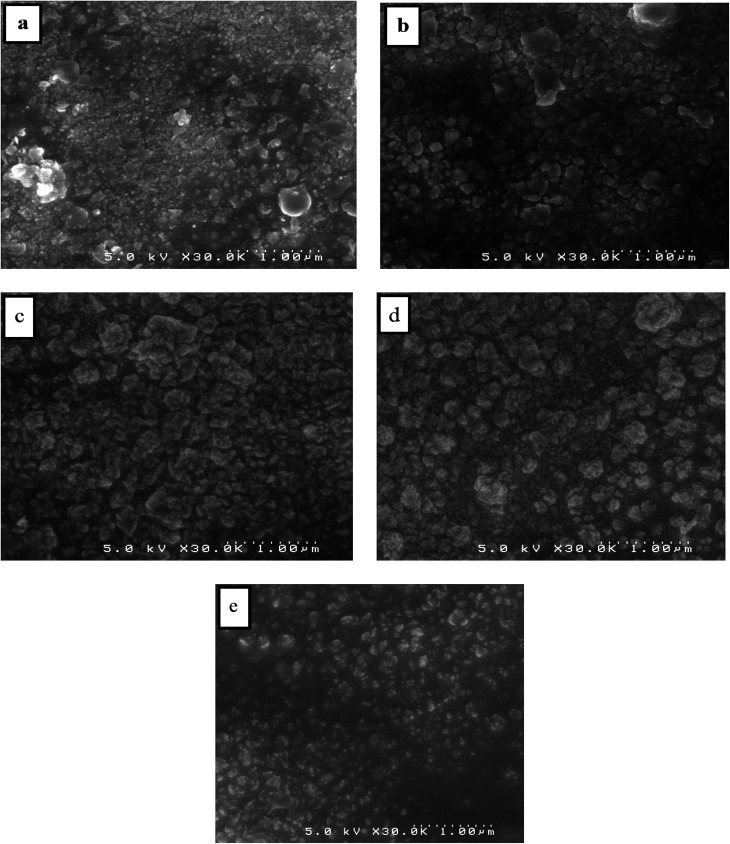
SEM images of (a) pure SnO_2_, (b) SnO_2_:Dy 1%, (c) SnO_2_:Dy 3%, (d) SnO_2_:Dy 5% and (e) SnO_2_:Dy 7% thin film samples.

SEM is used to analyze the surface morphology of pure and Dy-doped SnO_2_ thin films. The morphological modification in the SnO_2_ thin film samples at various doping rates (0, 1, 3, 5 and 7% at.) are shown in Fig. 5. These figures suggest that for pure SnO_2_, the grains do not seem to be tightly packed, leaving noticeable voids in the sample. Numerous microscopic grains multiplied and collected to create a grainy surface with the appearance of gaps and fissures as the rate of Dy increased at 1 and 3%. However, at a Dy rate equal to 5%, the surface of the chopped film is covered with numerous microaggregates that together create a granular and dense surface. In addition, small grains occurred between the aggregates to fill in the voids and condense the surface of the thin layer, indicating that the layer is well crystallized at this rate, which is also supported by the XRD data. The SEM photos show that the aggregation of these particles is evenly distributed for the SnO_2_:Dy 5% layers. As a result, charge carrier dispersion is reduced, increasing the electrical conductivity of films. The optoelectronic characteristics of our semiconductors may be enhanced by this behavior.^60^ The sample doped at Dy 5% exhibits the optimal stable properties; furthermore, the optical and structural results confirm this enhancement.

**Fig. 6:**
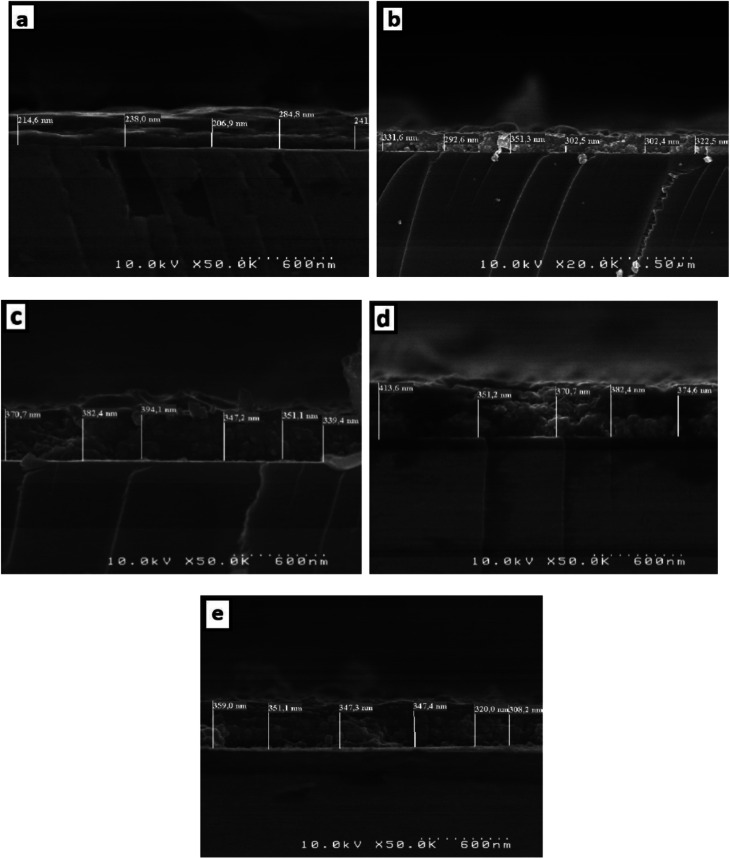
Cross-section of (a) pure SnO_2_, (b) SnO_2_:Dy 1%, (c) SnO_2_:Dy 3%, (d) SnO_2_:Dy 5% and (e) SnO_2_:Dy 7% thin film sample ESEM images.

The thickness of the thin films is obtained by measuring the side surface width in the cross section of an SEM image. Fig. 6 presents the SEM cross-section images for pure and doped SnO_2_ thin films. Using this method, the average thickness of the different SnO_2_ layers was determined to be 237, 317.5, 364, 378 and 339 nm for the 0, 1, 3, 5, and 7% Dy-doped thin films, respectively. In addition, the images allowed us to clearly see the effect of Dy, as the thickness value increases as the doping percentage increases. These results confirm those calculated by means of the interference fringes method only for the 7% Dy-doped SnO_2_ thin film, whose error range is slightly greater at about ±30 nm. This slight difference does not detract from the effectiveness of the interference fringes method and is related to the inhomogeneous character of the porous surfaces produced *via* the spray method.

### 3.4 Chemical composition

Energy-dispersive X-ray spectroscopy (EDS) is used to determine the chemical composition of the pure and Dy-doped SnO_2_ thin films. The elemental compositions obtained using EDS are shown in Table 3. Analysis of this data reveals the presence of Dy, Sn, and O elements in accordance with atomic ratios obtained from the initial precursors for respective samples, as well as Si (with substrate contribution) with very low concentration traces of Mg, Al, Na, Cl, and Ca.

**Table 3:** Elemental concentrations calculated from EDS of SnO_2_ thin films with different Dy levels

Spectrum	Sn	O	Dy	Si	Cl	Na	Mg	Al	Ca
SnO_2_	13.23 ± 0.60	57.23 ± 0.44	0.00	19.40 ± 0.66	1.57 ± 0.08	3.83 ± 0.29	1.07 ± 0.05	0.98 ± 0.03	2.67 ± 0.06
SnO_2_:Dy 1%	19.08 ± 0.79	56.10 ± 0.03	0.39 ± 0.01	14.68 ± 0.77	3.18 ± 0.40	2.33 ± 0.18	0.83 ± 0.06	1.06 ± 0.04	2.38 ± 0.10
SnO_2_:Dy 3%	21.27 ± 1.28	57.63 ± 0.23	0.95 ± 0.07	11.57 ± 1.4	3.55 ± 0.5	1.44 ± 0.24	0.58 ± 0.08	0.95 ± 0.01	2.06 ± 0.09
SnO_2_:Dy 5%	20.48 ± 1.23	57.67 ± 0.47	1.77 ± 0.11	11.90 ± 1.19	3.33 ± 0.25	1.32 ± 0.37	0.44 ± 0.17	0.92 ± 0.11	2.16 ± 0.12
SnO_2_:Dy 7%	20.78 ± 1.93	52.63 ± 0.78	2.58 ± 0.29	13.30 ± 1.92	5.43 ± 0.99	1.33 ± 0.57	0.36 ± 0.15	1.15 ± 0.13	2.44 ± 0.13

The correct levels of Dy are present in every layer. The high levels of O atoms are related to the experimental conditions, which included the use of H_2_O as a solvent.

### 3.5 Electrical properties

**Fig. 7:**
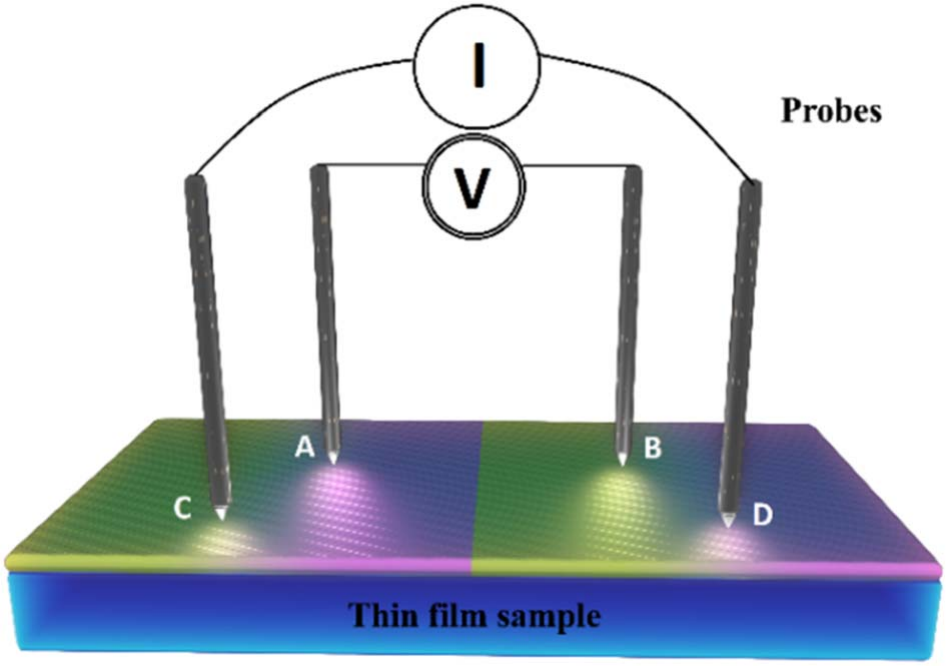
A diagram showing the four-point configuration used in taking the electrical measurements.

As illustrated in Fig. 7, a voltmeter was used to measure the voltage (*V*) across probes *A* and *B* while a high impedance (*I*) current source was utilized to deliver current through the two probes *C* and *D*. Eqn (13) was applied to the values of the observed voltages and sourced current to determine the bulk resistivity of the sample.^61^

13 *R*_b_ = *R*_s_ × *t*

where *t* is the thickness in cm of the contacted thin layer (calculated from the fringes in the reflection spectra) and *R*_s_ is sheet resistance for the thin films determined by the equation below:^62^14
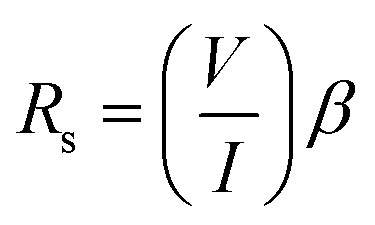
where *β* is a geometric factor attributed to 
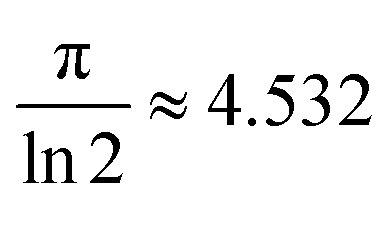
 in the case of semi-infinite thin films.

The electrical resistance and resistivity values are listed in Table 4.

**Table 4:** Electrical resistivity of the SnO_2_:Dy thin films with varying Dy dopant rates: 0, 1, 3, 5 and 7% at

Samples	Sheet resistance (Ω sq^−1^)	Electrical resistivity (×10^−4^ Ω cm)
SnO_2_ pure	42.76	102.07
SnO_2_:Dy 1%	25.11	95.41
SnO_2_:Dy 3%	18.73	69.88
SnO_2_:Dy 5%	10.46	33.49
SnO_2_:Dy 7%	87.05	330.16

**Fig. 8:**
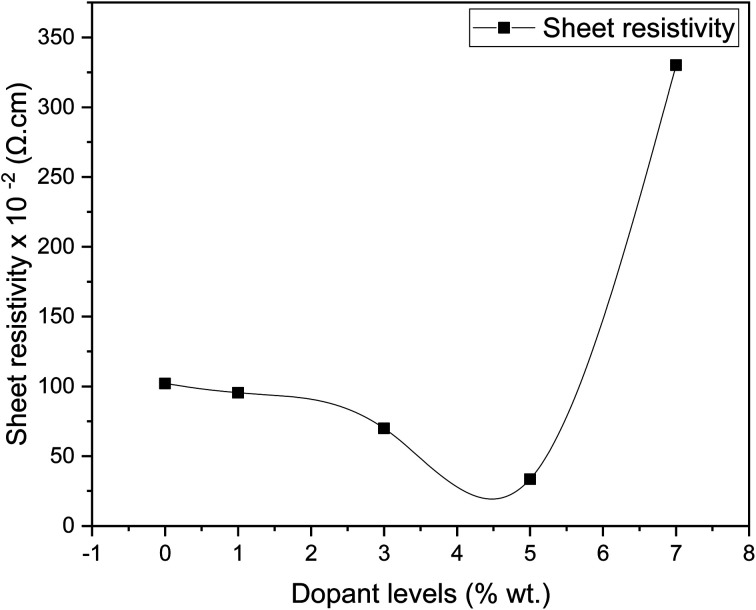
Variation of resistivity in sheet characteristics of SnO_2_:Dy by Dy levels.

Fig. 8 shows the sheet resistivity for pure SnO_2_ and Dy-doped thin films. All samples show low sheet resistivity, due to the formation of oxygen vacancies, which function as electron donors and raise the concentration of free carriers. The undoped SnO_2_ thin films showed a low sheet resistivity of 102.07 × 10^−4^ Ω cm, which can be attributed to the divergence from stoichiometry.^63^ First, the sheet resistivity dropped until it reached a minimal value of 33.49 × 10^−4^ Ω cm for the Dy 5% as the dopant concentration increased. This is due to the presence of Dy^3+^ ions in the SnO_2_ crystal lattice, leading to an addition in the numbers of the free electrons and causing an increase in the concentration of free carriers.^64^ The sheet resistivity of the thin films increased after the minimum point as the dopant concentration is raised above the ideal doping level. This is because the crystal structure of the thin film began to degenerate when the dysprosium atoms inserted themselves into the interstitial spaces, which resulted in a decrease in the mobility of free charge carriers and an increase in the sheet resistivity of the thin films. Similar results were obtained by Patrick and al., who examined the effect of Pd doping on SnO_2_ thin layers.^65^

### 3.6 Photocatalytic activity

The photocatalytic activity of the undoped and Dy-doped SnO_2_ thin films is evaluated using the degradation of methylene blue (MB). This organic dye exhibits a strong absorbance band at 660 nm whose intensity decreases as the MB degrades. Variations in intensity were assessed at regular time intervals. Fig. 11 shows the spectra of the degradation rate of MB after solar irradiation for three hours using the Dy-doped SnO_2_ thin films at various Dy concentrations under solar irradiation.

The diagrams in Fig. 14a demonstrate the good photocatalytic responses of pure and Dy-doped SnO_2_ thin films. MB photo degradation rates were about 85% at three hours under sunlight for all doped thin films. Furthermore, by calculating the degradation efficiency given by eqn (1), we were able to confirm the efficiency of the thin films through the degradation of the MB dye, which increased when the Dy doping level increased to reach 90.99% for 5% Dy, proving that the dopant caused the degradation mechanism. This effect is highest for the 5% doped thin films, which can be attributed to a higher number of free electrons (these samples show the highest conductivity) to powerfully activate the degradation activity, as well as to the band gap reaching the visible range, which enhanced the absorption of the sunlight irradiation. This is comparable to other results, and this low-cost approach could offer a flexible means of enhancing the photocatalytic activity of SnO_2_ to high levels of efficiency.

Table 5 provides some examples of the photocatalytic efficiency of SnO_2_, either doped or coupled with other materials, that has attracted the attention of many researchers:

**Table 5:** Comparison of dye efficiency in similar materials reported in the thin film literature

Coupled and doped catalyst	Light source/dye	Photodegradation efficiency	Ref.
SnO_2_:Zn	Visible annular brand photoreactor (UV)/MB	Efficiency ranges from 68 to 90%	66
SnO_2_/Fe	250 W UV light/rhodamine B	Degraded RhB solution by w55%	67
SnO_2_/Co	450 W Hg lamp/4-hydroxy benzoic acid	Complete photodegradation of 4-HBA	68
Graphene/SnO_2_	350 W Xe lamp sunlight/MB	Dye was degraded completely after 6 h	69
SnO_2_:Tb	UV lamp (*λ* = 254 nm)/(MB)	Efficiency goes from 72 to 85%	70
SnO_2_:Dy	Solar irradiation/MB	90.99%	Our work

To more clearly observe the effect of Dy doping on the photocatalytic activity of the thin films, the kinetic rate (*K*) is calculated using this formula:^71^15
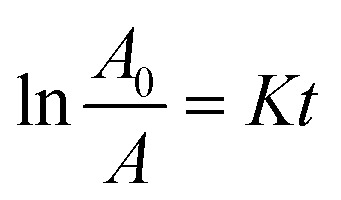
where *A*_0_ is the MB dye's starting absorbance, *A* is the dye's absorbance after exposure for time *t*, and *K* is a pseudo-first-order rate constant. The inset of Fig. 14b shows the analysis of the relationship between the degradation ratios ln(*A*/*A*_0_) of MB dye and time for both pure and doped SnO_2_.

The slope of the ln(*A*/*A*_0_) *vs.* irradiation time (*t*) plot yields the constant rate *K* (Fig. 14b). The results shows that the reaction rate constant increased as the doping concentration increased with a higher value of *K* for thin films doped at 5%, demonstrating that the addition of Dy to SnO_2_ at several levels improves photocatalytic activity under solar irradiation.

• Study of stability and reusability

**Fig. 9:**
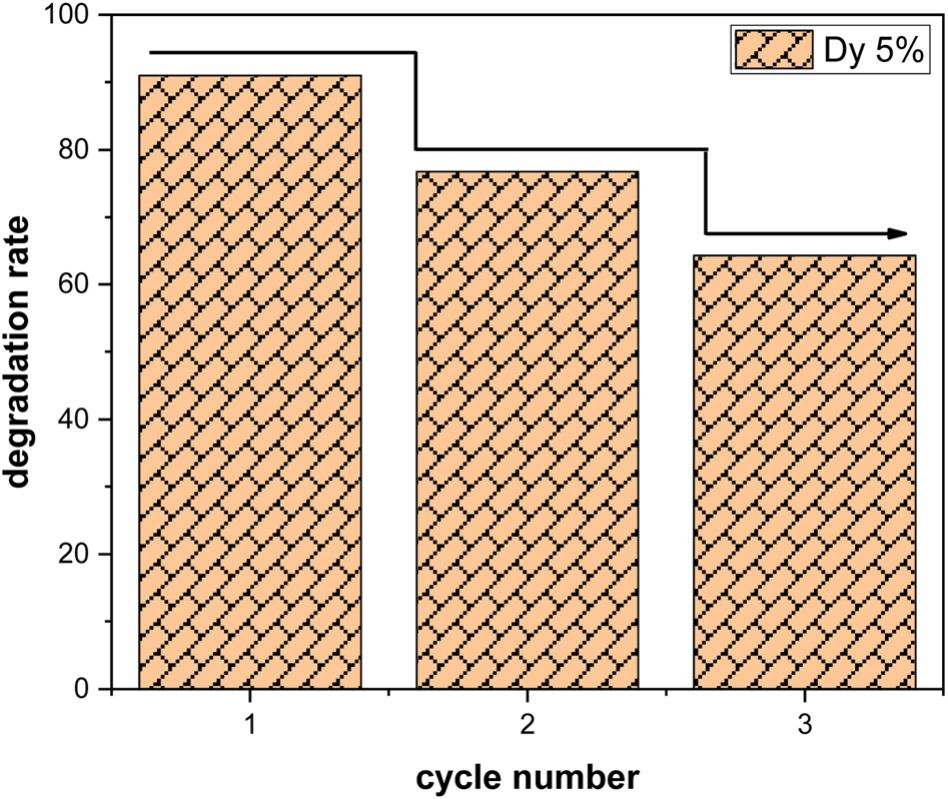
Recyclability of Dy doped SnO_2_ at 5%.

Three consecutive cycles of methylene blue (MB) degradation were carried out under solar irradiation, as depicted in the Fig. 9, to evaluate the sustained effectiveness of SnO_2_:Dy samples with a dye concentration of 3 mg L^−1^. After each cycle, the samples underwent a thorough washing process, involving multiple rinses with distilled water, followed by drying at 50 °C. Remarkably, only a minor decrease in photocatalytic efficiency, estimated at approximately 10%, is observed after each cycle. This highlights the remarkable resilience of Dy-doped SnO_2_ thin films in the degradation of methylene blue. It should be noted that the effectiveness of SnO_2_ in photocatalyzing methylene blue may vary depending on the specific experimental conditions or application.

• Effect of MB dye concentration

**Fig. 10:**
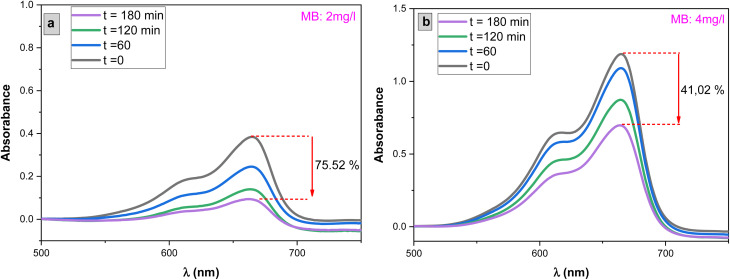
Degradation of MB dye *versus* irradiation time in the presence of sprayed SnO_2_:Dy [0, 1, 3, 5 and 7% at.] thin films with concentration of MB with MB concentrations (a) 2 mg L^−1^ and (b) 4 mg L^−1^.

**Fig. 11:**
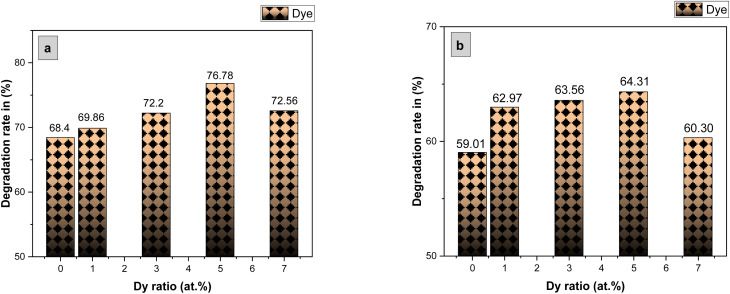
Degradation rate of MB in presence of the Dy-doped SnO_2_ thin films at various Dy ratios (0, 1, 3, 5 and 7% at.) for 3 hours under solar irradiation with MB concentrations (a) 2 mg L^−1^ and (b) 4 mg L^−1^.

The influence of methylene blue (MB) concentrations on the photocatalytic degradation process was investigated by varying MB concentrations from 2 mg L^−1^ to 4 mg L^−1^, with a 1 mg L^−1^ increment, while exposing the system to solar light irradiation as shown in Fig. 11. Fig. 10 illustrates the results. It was observed that the rate of MB degradation is highly efficient for concentrations up to 3 mg L^−1^. However, when the concentration exceeded 3 mg L^−1^ and reached 4 mg L^−1^, the rate of MB photodegradation slowed down, ultimately reaching only 41% degradation over a 3 hour period.This behavior can be attributed to increased competition between MB molecules for degradation and a decrease in the intensity of light reaching the surface of the SnO_2_ photocatalyst. At higher concentrations, the solution effectively screens a significant portion of the incoming light, allowing fewer photons to reach the SnO_2_ surface. Consequently, the production of electron–hole pairs, which are crucial for the degradation process, is significantly reduced. This reduction in electron–hole pairs, in turn, leads to a decrease in the degradation of the dye.^72^

• Mechanism of photocatalytic degradation

Much research has focused on better understanding the process of photocatalysis. It involves a sequence of reduction and oxidation reactions, where the electrons move from the valence band (VB) to the conduction band (CB), simultaneously generating oxidative valence holes (h^+^) and reductive conduction electrons (e), generally activated when the energy of the light irradiation is equal to or higher than the energy band gap of the catalyst (eqn (16)). In a redox reaction, which involves both electrons and holes, the excited conduction band electrons (e_CB_) are captured by oxygen molecules, creating reactive peroxide radicals (˙O_2_^−^) eqn (17). Hydroxyl radicals are then created when (h_VB_^+^) and the water (H_2_O) that has been adsorbed react on the surface of the thin film producing (OH˙), as shown in eqn (18).

Various dopants have been used to improve the effectiveness of photocatalytic reactions and decrease the process of hole and electron recombination. Rare earth dopants have been found to be one of the most effective materials for acting as trapping sites to slow down the recombination process and extend the mean lifetime.^73^ The doping procedure causes the trapped charges to be transported to the film's surface where they interact with the dye molecules to boost photocatalytic efficiency.^74^ In our case, dysprosium also has a potent 4f orbital that is partially occupied, making it a Lewis acid that may quickly react with electrons from the conduction band.^75^

According to eqn (24), a small number of (OH) radicals rearrange to create H_2_O_2_, which then dissociates into (OH) radicals as shown in eqn (25). Finally, the breakdown of the organic contaminants is demonstrated in eqn (26) by the hydroxyl radicals produced by the photocatalysis processes (Fig. 13).

16 SnO_2_ + *hν* → SnO_2_ (e_CB_^−^) + SnO_2_ (h_VB_^+^)

17 SnO_2_ (e_CB_^−^) + O_2_ → ˙O_2_^−^

18 O_2_^−^ + e^−^ + 2H^+^ → H_2_O_2_

19 Dy^3+^ + e_CB_^−^ → Dy_^2+^_

20 Dy^2+^ + O_2_(ads) → O^2−^

21 O^2−^ + H + → HO_2_

22 H_2_O_2_ + e^−^ → OH + OH^−^

23 SnO_2_ (h_VB_^+^) + H_2_O → ˙OH + H^+^

24 SnO_2_ + UV_irradiation_ + H_2_O → ˙H + ˙OH

25 H^+^ + OH˙ → HO˙

26 OH + methylene blue dye → CO_2_ + H_2_O + degraded products

**Fig. 12:**
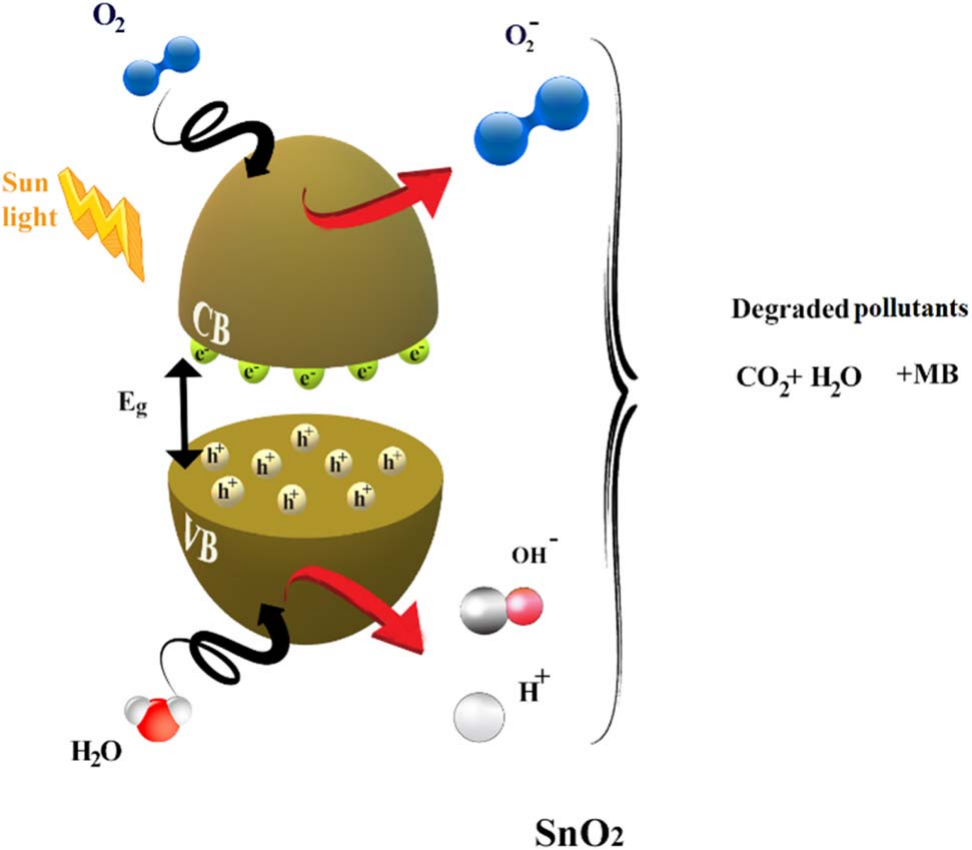
Mechanism of photodegradation of MB by SnO_2_ thin films under solar irradiation.

The mechanism of the photodegradation of MB is simplified in Fig. 12.

**Fig. 13:**
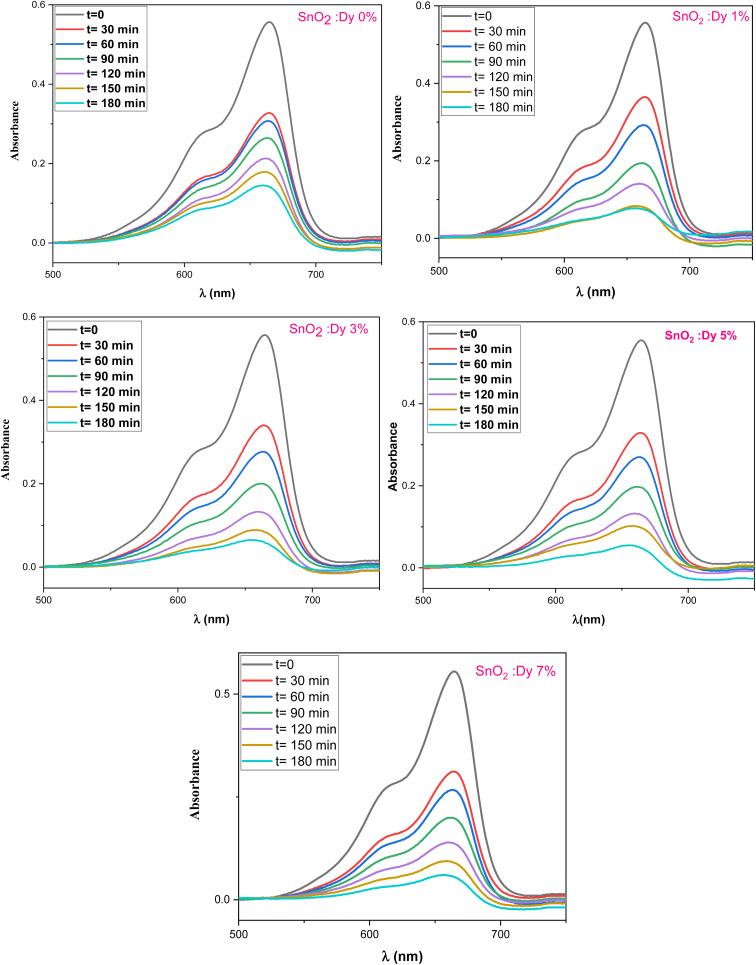
Degradation of MB dye *versus* irradiation time in the presence of sprayed SnO_2_:Dy [0, 1, 3, 5 and 7% at.] thin films.

**Fig. 14:**
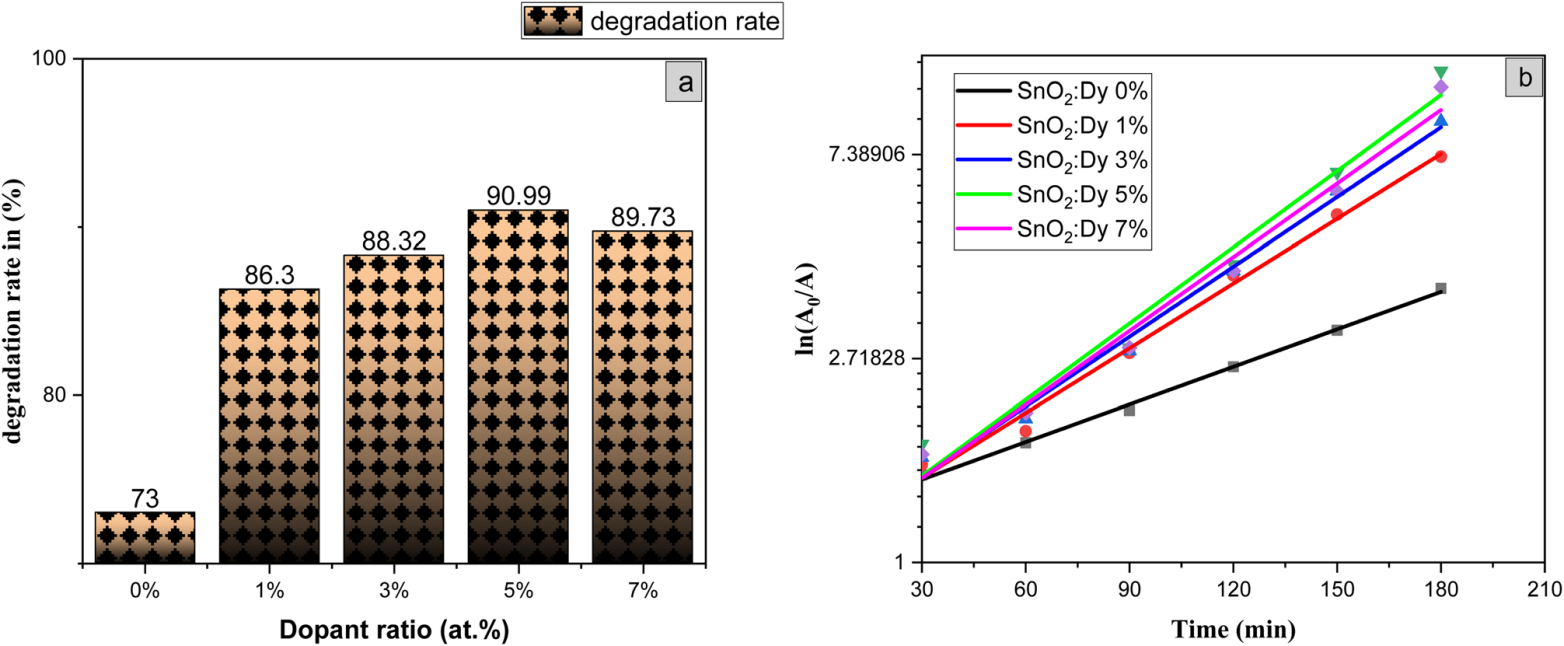
(a) Degradation rate of MB in presence of the Dy-doped SnO_2_ thin films at various Dy ratios (0, 1, 3, 5 and 7% at.) for 3 hours under solar irradiation, (b) graphic determination of the kinetic rate *K* as a function of Dy content.

Better photocatalytic activity can result from the overlap of a variety of factors like the higher electron mobility and charge carrier concentration of the samples and the right choice of doping materials at the right concentration levels. Other keys to achieving better results may include the synergistic effect on the adsorption phenomenon and the extended charge separation resulting from the interface of the photocatalyst species following charge transfer between two semiconducting materials, as well as intrinsic defects like oxygen vacancies that favor the interfacial charge transfer in their role as trapping centers of the photogenerated electrons and preventers of the electron–hole recombination.

## 4 Conclusions

Pure and dysprosium-doped SnO_2_ thin films were successfully synthesized on glass substrate using the spray pyrolysis technique. In order to obtain high-quality thin films, the impact of various dysprosium rates, which were examined by means of several different characterizations, was studied. XRD analyses revealed a tetragonal rutile structure for the SnO_2_:Dy thin films, with a better crystallization for the SnO_2_:Dy 5% thin films. The optical study showed that the materials used have high transparency levels and the optical gap decreases from 3.91 to 3.56 eV with an increase in the doping rate. SEM images showed an enhancement in the structure of the surface with an increase in the density of the grain to reach the maximum with SnO_2_:Dy 5%. In addition, sheet resistivity exhibited low values that decreased as the doping rates increased, reaching a minimum of 33.49 × 10^−2^ Ω cm for the 5% doped thin layers, which increased considerably for the 7% doped thin films. These results are confirmed by the destruction of the structure shown in the SEM images. Although methylene blue photodegradation showed high levels, reaching 90% in three hours under solar irradiation for the 5% Dy-doped SnO_2_ thin films, the cooperation of all these properties makes SnO_2_:Dy thin films good candidates for use in the field of photocatalytic applications such as wastewater treatment.

## Conflicts of interest

There are no conflicts to declare.

## Supplementary Material
